# IV-PAFNet: Infrared-visible cross-modal alignment and fusion network for nighttime UAV object detection

**DOI:** 10.1371/journal.pone.0361401

**Published:** 2026-10-28

**Authors:** Cheng Yuan, Shichang Shang, Jinghao Zhang, Yang Zhang, Bin Zhou

**Affiliations:** School of Computer Science and Technology, Shandong University of Technology, Zibo, Shandong, China; University of Electronic Science and Technology of China, CHINA

## Abstract

Nighttime unmanned aerial vehicle object detection is challenged by degraded visible-light textures, cluttered infrared thermal responses, and local spatial discrepancies between infrared and visible images. To address these issues, we propose IV-PAFNet, an infrared-visible purification, alignment, and fusion network designed for object detection in nighttime and low-visibility conditions. IV-PAFNet consists of two primary components. The infrared signal purification and spatial alignment module applies learnable high-pass filtering and gated residual fusion to refine infrared responses, followed by visible-guided offset prediction and differentiable resampling to alleviate local cross-modal spatial discrepancies. The multi-scale fusion attention enhancement module performs channel-wise modality recalibration and spatial reliability weighting at multiple feature-pyramid levels, allowing the network to adapt the contribution of each modality to local imaging conditions. In the training-based DroneVehicle and generalization-based Camera-vehicle1 datasets, IV-PAFNet achieves 62.81% mAP@0.5:0.95 and 51.4% mAP@0.5:0.95, respectively. Additional controlled experiments investigate the distinct roles of infrared purification, visible-guided alignment, channel recalibration, and spatial reliability modeling, while statistical and computational analyses characterize run-to-run variation and inference cost. The code is available at https://github.com/iv-pafnet/IV-PAFNet.

## Introduction

UAVs have been widely applied in scenarios such as security inspection, traffic monitoring, and emergency rescue due to their high mobility, low deployment cost, and wide observation range. Object detection is a fundamental task in UAV visual perception. In recent years, DINO, Co-DETR, and RT-DETR have continuously improved detection performance. Meanwhile, QETR and various feature alignment and multi-scale modeling methods designed for small object detection in remote sensing have further enhanced detection performance under complex backgrounds [1–7]. The VisDrone dataset and related studies based on DroneVehicle have further promoted the development of object detection in complex aerial imagery and infrared-visible UAV scenarios [8,9]. However, under nighttime or extremely low-illumination conditions, visible-light images are prone to noise, local overexposure, and large-area shadows, which significantly degrade the textures and edges of small objects such as vehicles and pedestrians. Infrared thermal imaging does not rely on external illumination and can provide relatively stable object contours, making it an important complementary modality for nighttime perception. In recent years, methods such as confidence-aware fusion, C2Former, and DAMSDet have shown that complementary modeling of infrared-visible modalities can improve detection robustness under complex illumination conditions [10–12]. However, the infrared modality also suffers from issues such as low-frequency thermal backgrounds and isolated thermal spots. If dual-modal features are directly concatenated, spurious thermal responses can be simultaneously amplified.

Along with modality-specific noise, cross-modal spatial discrepancies present another bottleneck in dual-modal UAV detection. Existing methods often assume that the two modalities are perfectly aligned. In practical flight scenarios, sensor baselines, platform vibrations, and attitude variations can cause local offsets between corresponding objects. Improper alignment may cause feature ghosting and localization drift, while learnable sampling provides a mechanism for adaptive cross-modal interaction. Moreover, illuminated regions may depend more on visible-light textures, whereas shadowed regions may rely more on infrared contours. A dynamic fusion strategy at the regional level is therefore required.

To address the above issues, we propose an infrared-visible cross-modal purification, alignment, and fusion network for nighttime low-visibility UAV object detection, termed IV-PAFNet. Unlike coarse strategies that rely on direct concatenation or fixed weights, IV-PAFNet decomposes cross-modal interaction into two main components: infrared refinement and visible-guided resampling. This process begins with the infrared signal purification and spatial alignment module (ISA), followed by multi-scale adaptive fusion through the multi-scale fusion attention enhancement module (MSFA). The main contributions are summarized as follows:

1. We propose an infrared signal purification and spatial alignment module (ISA) that combines learnable high-pass filtering, adaptive thermal-response gating, visible-guided offset prediction, and differentiable resampling. The module is designed to suppress redundant thermal responses and alleviate local cross-modal spatial discrepancies without assuming exact pixel-level registration.
2. We propose a multi-scale fusion attention enhancement module (MSFA) that performs channel-wise modality recalibration followed by spatial reliability modeling at each feature-pyramid level. This design permits the contributions of the infrared and visible modalities to vary across semantic channels and image regions.
3. IV-PAFNet achieves competitive performance on the training-based DroneVehicle dataset and the generalization-based Camera-vehicle1 dataset. Further ablation experiments validate the individual contributions of infrared purification, visible-light-guided alignment, channel recalibration, and spatial reliability modeling.

## Related work

### UAV object detection based on deep learning

UAV object detection is an important branch of machine vision in outdoor perception, with high application value in security inspection, traffic monitoring, and emergency response. Unlike conventional ground-level imagery, UAV aerial images exhibit top-down viewpoints, substantial scale variation, complex backgrounds, and densely distributed targets. General detectors often lack task-specific adaptation for aerial scenarios, which can lead to feature extraction failure, false detections, and missed detections.

Multi-scale feature fusion is a major direction for addressing scale variation in aerial images. Early detectors relied mainly on a single deep feature map, losing high-resolution shallow details and performing weakly on small objects. Methods based on Feature Pyramid Networks [13], multi-path aggregation, and bidirectional pyramid structures combine shallow spatial details with deep semantics. Deep-learning detectors are commonly divided into two-stage and one-stage paradigms [14]. Two-stage detectors, including Cascade R-CNN [15] and Faster R-CNN [16], separate proposal generation from classification and regression. One-stage detectors, represented by the YOLO series [17], formulate localization and classification as a unified regression problem and provide favorable inference efficiency.

Attention mechanisms are widely introduced into UAV detection frameworks to suppress background interference. Spatial and channel attention guide networks toward effective features, while deformable convolution and Transformer architectures capture wider contextual relationships [18]. DETR [19] reformulates object detection as a set-prediction task, enabling end-to-end training.

For dense and arbitrarily oriented objects, traditional anchor-based detectors require extensive manual hyperparameter tuning and can suffer from overlapping boxes after non-maximum suppression. Anchor-free methods such as CornerNet and CenterNet formulate detection as keypoint prediction and attribute regression [20,21]. Oriented bounding-box mechanisms have also been incorporated to improve localization for arbitrarily oriented aerial targets [22].

Current UAV detectors perform well under clear illumination but remain vulnerable to haze, rain, snow, and low-visibility conditions. These degradations reduce contrast, blur textures, and lower the signal-to-noise ratio, affecting feature extraction and localization [23,24]. The growing demand for all-weather UAV inspection and surveillance motivates more robust detection frameworks.

### UAV object detection in low-visibility conditions

Low-visibility conditions such as rain, snow, fog, and low illumination degrade aerial images and limit all-weather UAV perception. Existing research mainly follows three directions: image enhancement, end-to-end detection optimization, and cross-domain adaptation.

Image preprocessing attempts to restore degraded images before detection. Classical methods include dark-channel-prior dehazing [25] and temporal-chromatic rain removal [26]. Deep-learning approaches use generative adversarial networks [27] and style transfer [28]. However, an enhance-then-detect pipeline may over-smooth images or remove small-object details.

End-to-end approaches embed anti-interference modules into detection networks. Transformer-based frameworks use dynamic contrast modeling [29], while skip connections and multi-scale fusion compensate for detail loss [30]. Dual-attention networks [31] and hyper-feature pyramids [32] can suppress background noise but may introduce substantial computational cost.

Unsupervised domain adaptation transfers knowledge from clear images to degraded conditions. TTSDA-YOLO uses multi-stage training to align feature distributions [33], and teacher-student strategies generate pseudo-labels for unlabeled degraded images. Nevertheless, coarse feature alignment may fail to distinguish weather noise from subtle target features.

### Infrared-visible cross-modal fusion object detection

When visible-light sensing becomes unreliable under nighttime illumination or heavy haze, single-modality detection cannot provide stable performance. Infrared-visible cross-modal detection uses the complementary properties of the two modalities to improve robustness.

Visible images provide texture, color, and fine detail, while infrared images are less dependent on ambient illumination and emphasize thermal contours. Existing studies have investigated cross-modal interaction, feature alignment, and large-scale multimodal benchmarks.

Many fusion methods assume strict spatial alignment. In UAV scenarios, sensor-baseline offsets, vibration, and pose variation can cause local translation, scale, and orientation differences. Direct fusion may therefore produce ghosting and boundary distortion. Learnable sampling and deformable attention provide mechanisms for reducing the influence of such discrepancies [3,30,34].

Modality reliability also varies across scenes and regions. Visible features are generally more reliable in well-lit areas, whereas infrared features may be more informative in shadowed regions. Fixed-weight fusion cannot adapt to this variation. The remaining challenge is therefore to refine noisy infrared responses, reduce the effect of local cross-modal discrepancy, and allocate modality weights according to local conditions.

## Method

As shown in Fig 1, IV-PAFNet contains two core modules: the Infrared Signal Purification and Spatial Alignment module (ISA), which refines infrared responses and performs visible-guided resampling before fusion; and the Multi-Scale Fusion Attention Enhancement module (MSFA), which conducts adaptive heterogeneous feature fusion at each feature-pyramid level. The fused features are then optimized through the detection loss.

**Fig 1:**
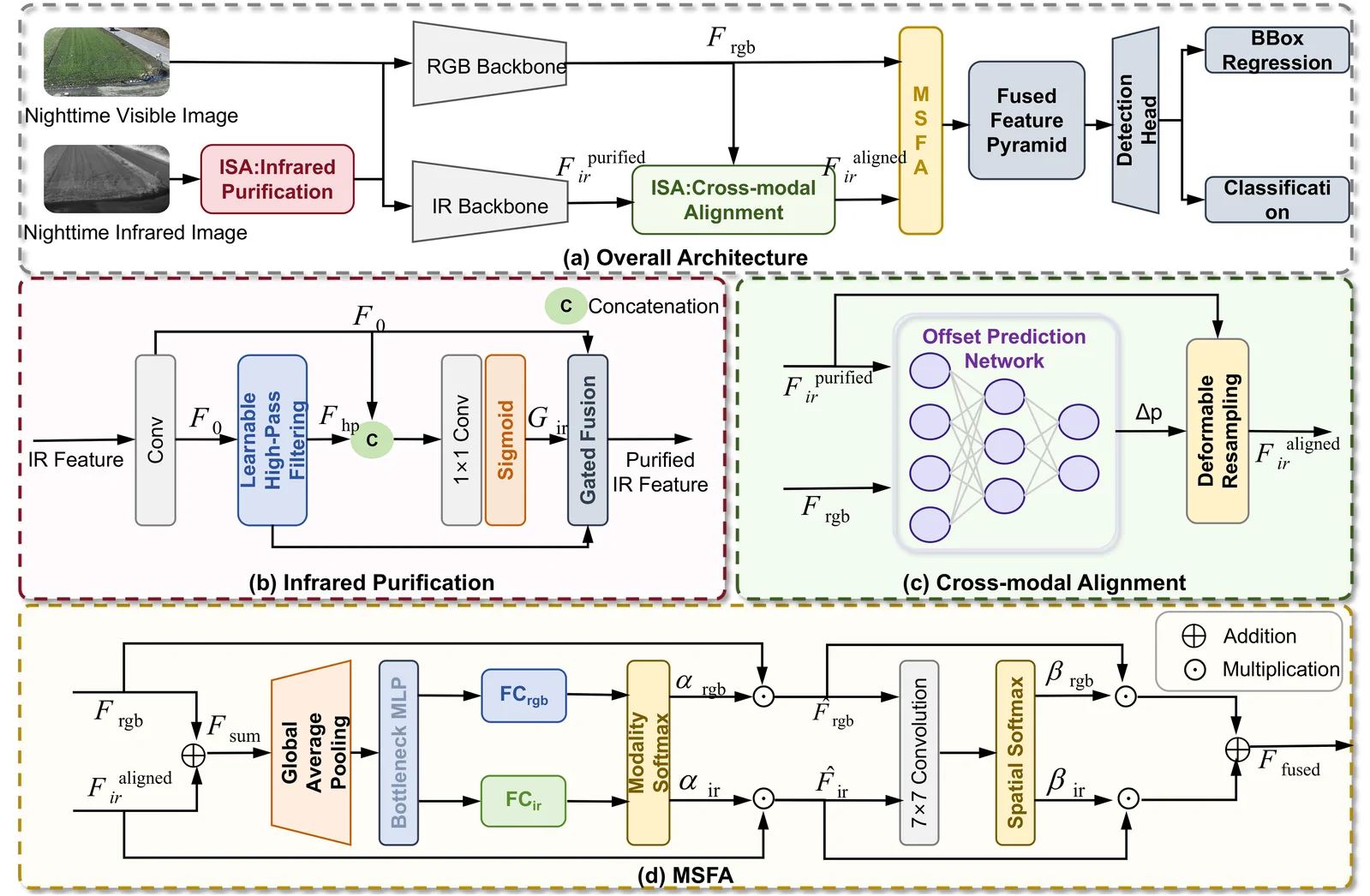
Framework of the proposed IV-PAFNet. (a) Overall architecture. (b) Infrared purification of ISA. (c) Cross-modal alignment of ISA. (d) MSFA. The sample images in the figure are only for illustration purposes. The representative visible and infrared samples are from the 3D-Net dataset that follows the CC BY 4.0 license.

### Infrared signal purification and spatial alignment

ISA adopts a two-step sequence for modality purification and visible-guided resampling to address infrared thermal-background interference and reduce the effect of local cross-modal discrepancy. The output is denoted as the aligned infrared feature $F_{ir}^{aligned}$.

#### 1) Infrared thermal-response purification.

Given the infrared image $X_{ir}$, a shallow convolution first maps the input into the feature space:


$$\begin{array}{c} F_{0}={𝒞}_{s}(X_{ir}), \end{array}$$ (1)

Here, ${𝒞}_{s}(\cdot )$ denotes the shallow convolutional mapping function, and *H*, *W*, and *C* denote the height, width, and number of channels of the feature map. A learnable high-pass filter is then used to extract high-frequency detail responses:


$$\begin{array}{c} F_{hp}=W_{hp}*F_{0}+b_{hp}, \end{array}$$ (2)

The learnable kernel $W_{hp}$ is initialized with a classical high-pass operator, such as a Laplacian kernel, and is subsequently optimized end-to-end. To distinguish target edges from isolated thermal spots, *F*_0_ and $F_{hp}$ are concatenated and passed through a 1×1 convolution followed by a sigmoid function:


$$\begin{array}{c} G_{ir}=\sigma ({\mathrm{Conv}}_{1\times 1}([F_{0},F_{hp}])), \end{array}$$ (3)

The confidence map $G_{ir}$ controls gated fusion between the high-frequency response and the initial structure:


$$\begin{array}{c} F_{ir}^{purified}=G_{ir}⊙F_{hp}+(1-G_{ir})⊙F_{0}, \end{array}$$ (4)

where ⊙ denotes the element-wise Hadamard product. This mechanism enhances the thermal contours of objects while suppressing redundant thermal backgrounds and isolated thermal spots. The purified feature $F_{ir}^{purified}$ is subsequently fed into the infrared backbone for multi-scale feature extraction. For brevity, the resulting backbone feature is still denoted as $F_{ir}^{purified}$.

#### 2) Cross-modal flexible alignment.

The alignment stage follows the learnable-sampling concept from deformable convolution [30]. Unlike standard deformable convolution, which predicts offsets from a single modality, this stage uses the visible feature $F_{rgb}$ as the reference and predicts infrared sampling offsets from the concatenated dual-modal representation:


$$\begin{array}{c} \Delta P={\mathrm{Conv}}_{offset}([F_{rgb},F_{ir}^{purified}]), \end{array}$$ (5)

${\mathrm{Conv}}_{offset}$ consists of two groups of 3×3 depthwise convolutional layers with nonlinear activations. ΔP encodes two-dimensional offsets for *N* sampling points at each location. Because the predicted coordinates may be non-integer, bilinear interpolation is used for differentiable sampling:


$$\begin{array}{c} F_{ir}^{aligned}(p_{0})=\sum_{n=1}^{N}w_{n}\cdot Bilinear\;\left[F_{ir}^{purified}\right]\;(p_{0}+p_{n}+\Delta p_{n}), \end{array}$$ (6)

where $p_{n}$ denotes the *n*-th regular reference sampling position in the standard convolution grid, and $w_{n}$ denotes the learnable spatial aggregation weight. The operation is intended to reduce the effect of local spatial discrepancy relative to the visible reference while retaining infrared thermal information.

### Multi-scale fusion attention enhancement

MSFA is embedded at multiple feature-pyramid levels and follows a coarse-to-fine attention sequence. Channel-level recalibration estimates the relative contribution of each modality for each semantic channel, while spatial reliability modeling assigns complementary local weights after channel recalibration. The formulation below describes one scale.

#### 1) Channel-level modality recalibration.

The two modalities are first aggregated as $F_{sum}=F_{rgb}+F_{ir}^{aligned}$. Global average pooling and a bottleneck multilayer perceptron with reduction ratio *r* produce a channel descriptor:


$$\begin{array}{c} z={\mathrm{MLP}}_{s}(\mathrm{GAP}(F_{sum})), \end{array}$$ (7)

The descriptor is passed to two independent fully connected layers and normalized along the modality dimension:


$$\begin{array}{c} {}[\alpha_{rgb},\alpha_{ir}]={\mathrm{Softmax}}_{m}({\mathrm{FC}}_{rgb}(z),{\mathrm{FC}}_{ir}(z)), \end{array}$$ (8)

The modality weights satisfy $\alpha_{rgb}^{\left(c\right)}+\alpha_{ir}^{\left(c\right)}=1$ for each channel. The recalibrated features are ${\hat{F}}_{rgb}=\alpha_{rgb}⊙F_{rgb}$ and ${\hat{F}}_{ir}=\alpha_{ir}⊙F_{ir}^{aligned}$.

#### 2) Spatial-level reliability selection.

The recalibrated features are concatenated and processed by a 7×7 convolution followed by a softmax operation:


$$\begin{array}{c} {}[\beta_{rgb},\beta_{ir}]={\mathrm{Softmax}}_{c}({\mathrm{Conv}}_{7\times 7}([{\hat{F}}_{rgb},{\hat{F}}_{ir}])), \end{array}$$ (9)

The complementary gates satisfy $\beta_{rgb}+\beta_{ir}=1$ and model spatially varying modality reliability.

#### 3) Dual-weighted fusion.

Channel-level and spatial-level weights are jointly applied to obtain the fused output:


$$\begin{array}{c} F_{fused}=\beta_{rgb}⊙{\hat{F}}_{rgb}⊕\beta_{ir}⊙{\hat{F}}_{ir}. \end{array}$$ (10)

### Detection loss

The fused feature pyramid is fed into the detection head for class prediction and bounding box regression. A multi-task joint loss is adopted, consisting of a weighted combination of the classification loss, the basic bounding box regression loss, and the generalized intersection over union loss:


$$\begin{array}{c} ℒ=\lambda_{cls}{ℒ}_{cls}+\lambda_{box}{ℒ}_{1}+\lambda_{GIoU}{ℒ}_{GIoU}, \end{array}$$ (11)

where $\lambda_{cls}$, $\lambda_{box}$, and $\lambda_{GIoU}$ denote the corresponding weighting coefficients. This joint loss enables the ISA and MSFA modules to be collaboratively optimized end-to-end under the supervision of the final detection objective.

## Experimental results

### Datasets and annotation protocol

The evaluation uses DroneVehicle and Camera-vehicle1 as the main benchmarks. DroneVehicle [9] contains 28,439 paired UAV-view visible and infrared images with five vehicle categories: car, truck, bus, van, and cargo vehicle. We follow the official split of 17,990 training pairs, 1,469 validation pairs, and 8,980 testing pairs. Because the compared detectors output horizontal bounding boxes, the original oriented annotations are converted into their minimum enclosing horizontal rectangles for both training and evaluation.

Camera-vehicle1 contains 12,483 paired self-made infrared-visible images collected by ground cameras in parking lots, highways, and urban streets. It serves as a cross-scene benchmark for evaluating generalization under changes in viewpoint and background distribution. Following the established evaluation protocol, models trained on DroneVehicle are evaluated directly on Camera-vehicle1 without fine-tuning for the target domain.

### Implementation details and evaluation metrics

All experiments are implemented using PyTorch. Input visible-infrared pairs are resized to 640×640. The released IV-PAFNet, MMPedestron [35], and component configurations use 73 epochs with the AdamW optimizer, cosine-annealing learning-rate scheduling, exponential moving average, and automatic mixed precision. Unless otherwise specified, the batch size is set to 8. The evaluation metrics include Precision, Recall, F1-score, mAP@0.5:0.95 averaged over IoU thresholds from 0.5 to 0.95, and mAP@0.5 following standard object-detection protocols [36,37]. For MMPedestron and IV-PAFNet, mAP@0.5:0.95 is reported as the mean ± sample standard deviation across seeds 42, 43, and 44. Precision, Recall, F1-score, and mAP@0.5, along with controlled component ablations, are reported for seed 43, which achieved the highest IV-PAFNet mAP@0.5 among the evaluated seeds. The same seed is used for the corresponding controlled-baseline comparisons. The accompanying reproducibility package provides the training, evaluation, preprocessing, and dataset-conversion scripts; the 73-epoch configurations; and environment specifications needed to rerun this protocol.

### Comparison with representative methods

We compare IV-PAFNet with three categories of representative detectors: classical CNN-based detectors, including ATSS [38], YOLOv7 [39], YOLOv8 [40] and YOLOv12 [41]; Transformer-based detectors, including Deformable DETR [30], RT-DETR [3], DINO [1], Co-DETR [2], and the multimodal detector MMPedestron [35]; and Mamba-based detectors, including VMamba [42], Mamba-YOLO [43], and AMSRDet [44]. Single-modal methods use early-fused infrared-visible images (6-channel concatenation) as input.

Table 1 compares IV-PAFNet with representative CNN, Transformer, and Mamba-based detectors on DroneVehicle. The released 73-epoch protocol applies to IV-PAFNet, MMPedestron, and the component variants. IV-PAFNet achieves 88.50% precision, 81.40% recall, 84.80% F1-score, 62.81±0.70% mAP@0.5:0.95, and 85.64% mAP@0.5. Compared with MMPedestron, IV-PAFNet improves mean mAP@0.5:0.95 by 0.80 percentage points and mAP@0.5 by 1.44 percentage points, while also achieving higher precision, recall, and F1-score.

**Table 1:** Comparison of multimodal object detection results on DroneVehicle.

Method	Type	P (%)	R (%)	F1 (%)	mAP@0.5:0.95 (%)	mAP@0.5(%)
ATSS	CNN	65.8	61.2	63.4	41.3	72.8
YOLOv7	CNN	70.5	65.8	68.1	43.6	76.8
YOLOv8	CNN	72.1	67.3	69.6	44.2	78.2
YOLOv12	CNN	73.6	69.2	71.3	45.1	79.8
Deformable DETR	Transformer	71.3	66.2	68.7	43.2	76.2
RT-DETR	Transformer	73.4	68.5	70.9	44.7	79.1
DINO	Transformer	74.2	69.1	71.6	45.3	80.2
Co-DETR	Transformer	73.9	69.8	71.8	45.1	80.4
MMPedestron	Transformer	84.20	79.50	81.78	62.01	84.20
VMamba	Mamba-based	71.6	67.4	69.4	43.7	77.1
Mamba-YOLO	Mamba-based	72.4	68.2	70.2	44.3	78.4
AMSRDet	Mamba-based	75.6	71.2	73.3	45.8	81.2
**IV-PAFNet**	**Transformer**	**88.50**	**81.40**	**84.80**	**62.81**	**85.64**

Table 2 reports cross-scene results on Camera-vehicle1 without target-domain fine-tuning. Under this evaluation, IV-PAFNet obtains 51.4% mAP@0.5:0.95 and MMPedestron obtains 48.2%, a difference of 3.2 percentage points.

**Table 2:** Comparison of cross-scene detection results on Camera-vehicle1 without target-domain fine-tuning.

Method	Type	P (%)	R (%)	F1 (%)	mAP@0.5:0.95 (%)
MMPedestron	Transformer	75.2	70.1	72.4	48.2
IV-PAFNet	Transformer	78.9	72.3	75.4	51.4

### Statistical analysis

To evaluate the robustness of the results against random initialization, MMPedestron and IV-PAFNet are each trained three times using different random seeds. As shown in Table 3, the controlled baseline achieves 62.01±0.42% mAP@0.5:0.95, while IV-PAFNet obtains 62.81±0.70% mAP@0.5:0.95. IV-PAFNet outperforms the baseline across all three seeds and improves mean mAP@0.5:0.95 by 0.80 percentage points. The reported standard deviations quantify the remaining run-to-run variation.

**Table 3:** Statistical analysis under different random seeds on DroneVehicle.

Method	Seed 42 mAP@0.5:0.95 (%)	Seed 43 mAP@0.5:0.95 (%)	Seed 44 mAP@0.5:0.95 (%)	Mean ± Std (%)
Baseline (MMPedestron)	61.98	62.44	61.60	62.01±0.42
IV-PAFNet	62.63	63.59	62.22	62.81±0.70

### Fine-grained component ablation

All component variants are evaluated with seed 43 under the same training protocol, enabling controlled comparison with the corresponding seed-43 baseline. Table 4 further examines the individual roles of infrared purification and visible-guided alignment. These results show that the purification‑only and alignment‑only variants have limited effectiveness when used in isolation, whereas the complete ISA configuration achieves the highest mAP@0.5:0.95. This pattern indicates that purification and alignment are more effective when combined sequentially, rather than suggesting that either component is ineffective in general. The results provide downstream task-level evidence of complementarity, but they do not constitute a direct geometric measurement of alignment quality. Full ISA reaches 84.18% mAP@0.5, below the complete IV-PAFNet result of 85.64%.

**Table 4:** Component-wise ablation of ISA on DroneVehicle.

Variant	Purification	Alignment	mAP@0.5:0.95 (%)	mAP@0.5 (%)
Baseline (MMPedestron)	–	–	61.98	84.20
Purification only	Yes	–	58.82	81.97
Alignment only	–	Yes	60.63	82.64
Full ISA	Yes	Yes	62.19	84.18

Table 5 separates channel recalibration and spatial reliability modeling. The spatial-only variant reaches 62.00% mAP@0.5:0.95, whereas the channel-only variant performs below MMPedestron. Full MSFA obtains 62.00% mAP@0.5:0.95 and 85.50% mAP@0.5. Its mAP@0.5:0.95 is comparable to that of the spatial-only variant. Meanwhile, its mAP@0.5 is 1.30 percentage points above the baseline but remains 0.14 percentage points below the complete IV-PAFNet result of 85.64%. The results do not support a claim that channel recalibration independently improves every metric.

**Table 5:** Component-wise ablation of MSFA on DroneVehicle.

Variant	Channel recalibration	Spatial reliability	mAP@0.5:0.95 (%)	mAP@0.5 (%)
Baseline (MMPedestron)	–	–	61.98	84.20
Channel only	Yes	–	60.30	82.70
Spatial only	–	Yes	62.00	83.90
Full MSFA	Yes	Yes	62.00	85.50

### Parameter sensitivity analysis

We examine two key hyperparameters: the channel reduction ratio *r* in the MSFA module and the geometric loss weight $\lambda_{GIoU}$ in the loss function. The parameter *r* determines the compression width of the bottleneck-style multilayer perceptron. If *r* is too small, the compressed descriptor has insufficient representational capacity, whereas an overly large *r* introduces redundant parameters. As shown in Fig 2, the experimental results show that the mAP reaches 62.81% when *r* = 16, achieving the optimal balance. We further sweep $\lambda_{GIoU}$ in the range of [1.0, 3.0] with a step size of 0.5. An excessively large weight over-constrains the geometric error and thus compromises classification optimization, while an overly small weight weakens localization accuracy. As shown in Fig 3, the performance peaks at $\lambda_{GIoU}=2.0$; therefore, this setting is adopted in our method.

**Fig 2:**
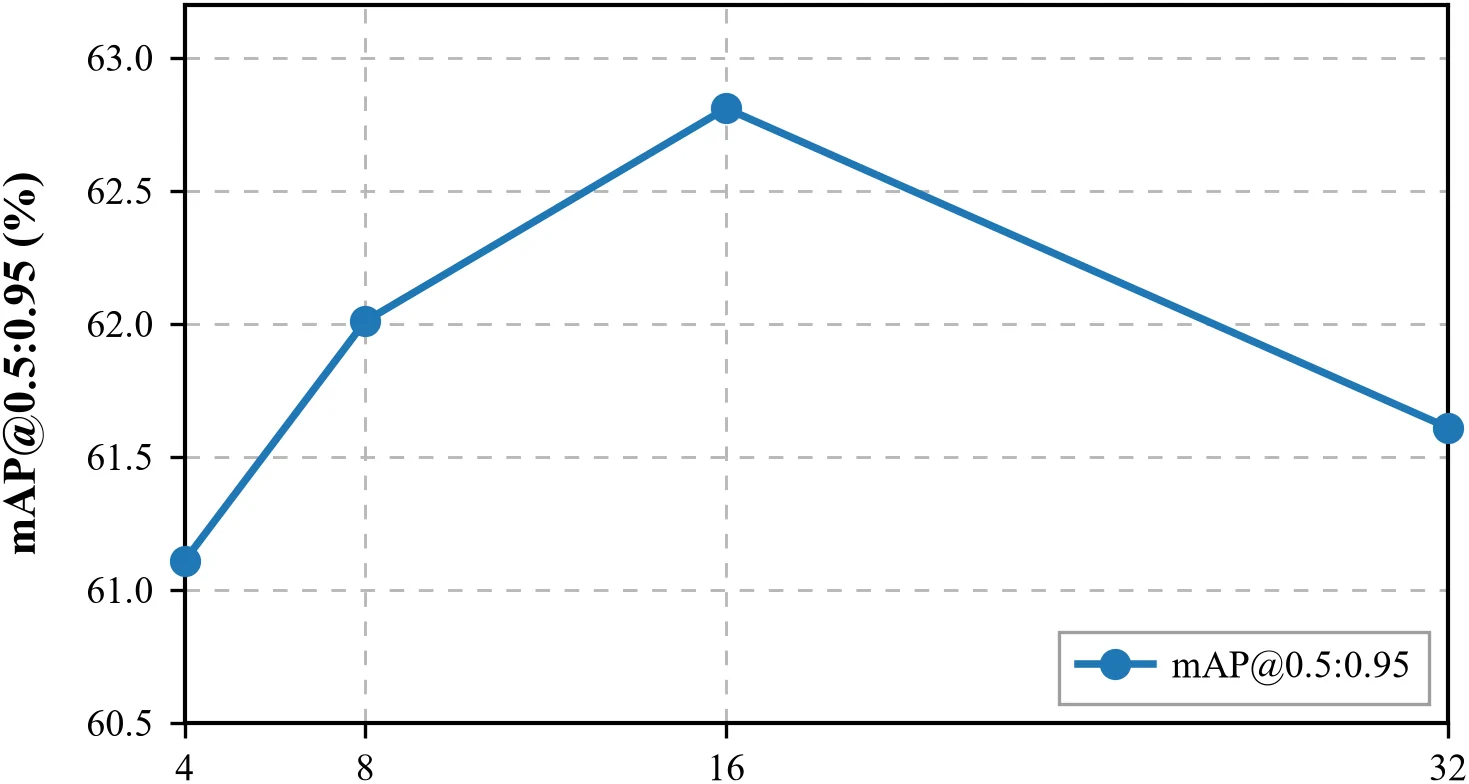
Sensitivity analysis of the channel-reduction ratio *r* in MSFA on DroneVehicle.

**Fig 3:**
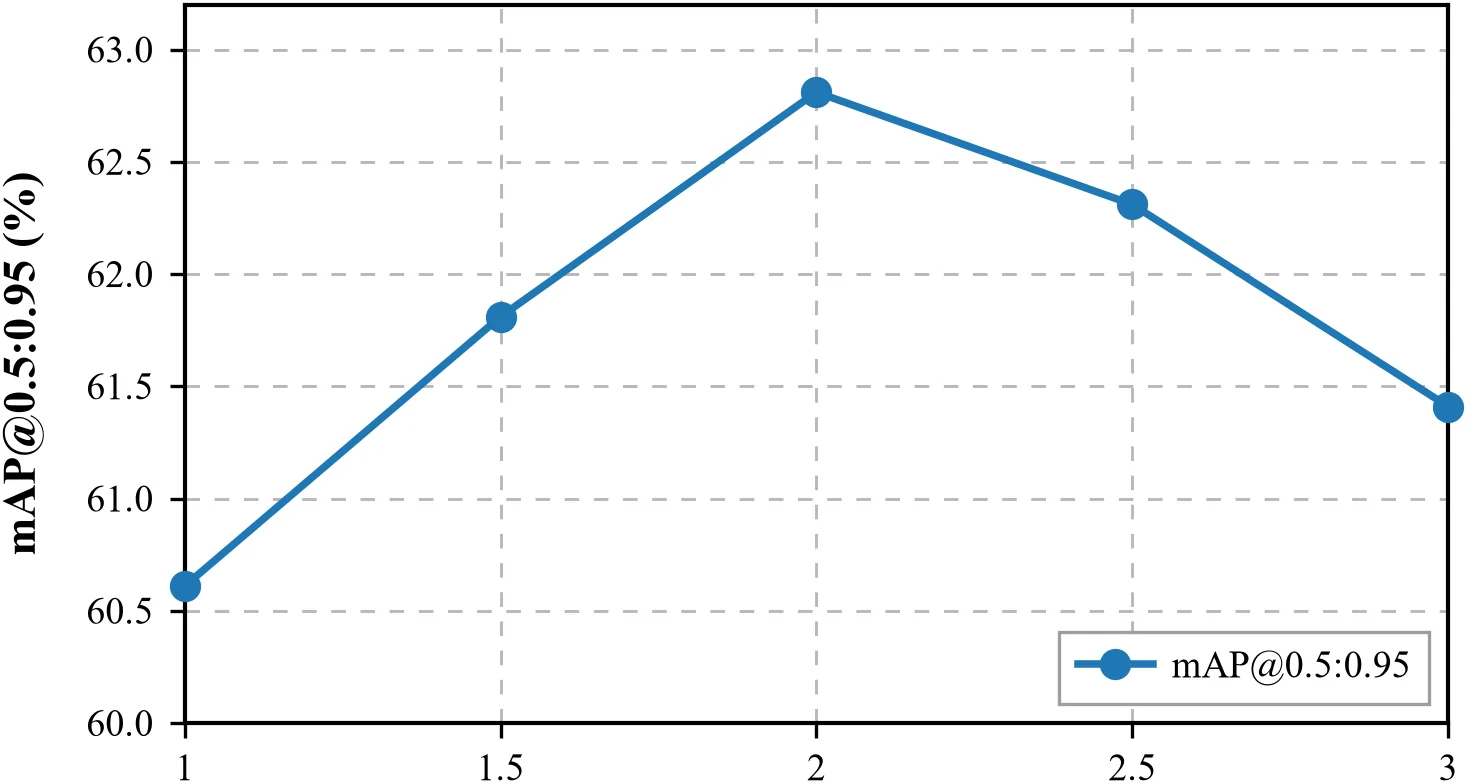
Sensitivity analysis of the geometric-loss weight $\lambda_{GIoU}$ on DroneVehicle.

### Computational complexity analysis

Table 6 summarizes computational cost and inference efficiency. All efficiency measurements use batch size 1 and a paired 640×640 visible-infrared input. IV-PAFNet increases the parameter count from 43 M to 52 M and FLOPs from 125 G to 154 G. Peak inference memory, defined as the maximum allocated CUDA memory during inference, increases from 3.4 GiB to 3.8 GiB. Throughput decreases from 26 FPS to 23 FPS, corresponding to latency values of 38.46 ms and 43.48 ms, respectively. Latency includes forward propagation and model-specific post-processing but excludes image loading, disk I/O, and visualization, and is calculated from the measured FPS as 1000/FPS. The additional costs are primarily associated with infrared purification, offset prediction, differentiable resampling, and multi-scale attention. These results indicate a moderate computational trade-off rather than a lightweight design.

**Table 6:** Computational complexity and inference efficiency.

Method	Params (M)	FLOPs (G)	Peak memory (GiB)	Latency (ms)	FPS
Baseline (MMPedestron)	43	125	3.4	38.46	26
IV-PAFNet	52	154	3.8	43.48	23

## Discussion

The experiments provide a nuanced interpretation of the proposed modules. The complete model achieves competitive performance on DroneVehicle and Camera-vehicle1, and the component-level ablations clarify how ISA and MSFA contribute to the final detector. For ISA, the purification-only and alignment-only variants do not individually reach the performance of the complete ISA configuration, while the full ISA achieves the highest mAP@0.5:0.95 among the ISA variants. This supports an interpretation of sequential complementarity: infrared refinement and visible-guided resampling are most effective when applied together, rather than as isolated operations.

For MSFA, spatial reliability modeling contributes more consistently than channel recalibration in isolation. Full MSFA improves mAP@0.5 relative to MMPedestron, while its mAP@0.5:0.95 remains close to the spatial-only result. Its mAP@0.5 of 85.50% remains 0.14 percentage points below the complete IV-PAFNet result of 85.64%. These results indicate that the two attention branches do not improve every metric independently, and that their contribution depends on their interaction with the complete architecture.

The statistical analysis further shows that IV-PAFNet outperforms MMPedestron across all three random-seed runs. Across three random seeds, IV-PAFNet achieves 62.81±0.70% mAP@0.5:0.95 compared with 62.01±0.42% for the baseline, corresponding to a mean improvement of 0.80 percentage points.

The efficiency analysis reports additional parameters, FLOPs, memory use, and latency for IV-PAFNet. Table 6 shows lower throughput for IV-PAFNet alongside its higher detection metrics in the evaluated setting.

The evaluation covers two complementary settings. DroneVehicle contains paired infrared-visible UAV images focused on vehicle categories, while Camera-vehicle1 provides a cross-scene test with paired infrared-visible images. Together, they assess changes in viewpoint and background distribution. Evaluation on additional multispectral UAV datasets spanning other platforms, sensor responses, regions, object categories, and weather conditions would further characterize transfer beyond the present data distributions.

IV-PAFNet assumes synchronized infrared-visible input pairs. Its behavior under sensor dropout, large synchronization errors, calibration changes, and thermal crossover remains to be characterized. ISA is currently evaluated through downstream detection metrics: the ablations show the contribution of visible-guided resampling within the complete detector, while controlled spatial perturbations and correspondence-based metrics could additionally quantify residual registration error and the range of correctable displacement.

Finally, the three-seed experiments quantify run-to-run variation under the reported training setting. IV-PAFNet improves mean mAP@0.5:0.95 by 0.80 percentage points on DroneVehicle; additional initializations, data splits, and external datasets would refine the estimate of this difference. ISA and MSFA also increase parameters, FLOPs, memory use, and latency. The reported 23 FPS measures model inference and excludes image loading, disk I/O, and visualization, so end-to-end onboard performance should be measured on the target UAV platform. Future work will evaluate missing or corrupted modalities, quantify alignment under controlled offsets, and investigate lightweight designs and onboard deployment.

## Conclusion

We propose IV-PAFNet for nighttime infrared-visible UAV object detection on UAV-captured imagery. ISA integrates learnable infrared-response refinement with visible-guided resampling, while MSFA emphasizes channel and spatial modality weighting across the feature pyramid. Under the released 73-epoch configurations, IV-PAFNet achieves 62.81 ± 0.70% mAP@0.5:0.95 across three seeds and 85.64% mAP@0.5 in the best-performing seed-43 run on DroneVehicle, as well as 51.4% mAP@0.5:0.95 on Camera-vehicle1. The component-level experiments show that infrared purification and alignment are most useful when combined, while combining spatial reliability modeling with channel recalibration also produces effective results. The computational analysis identifies a moderate cost increase. Future work will focus on direct alignment-quality evaluation and lightweight multimodal interaction for improved robustness across scenes.
