# Supplementary material for: IV-PAFNet: Infrared-visible cross-modal alignment and fusion network for nighttime UAV object detection
Source: PLoS One. 2026 Oct 28;21(10):e0361401. doi: 10.1371/journal.pone.0361401 (PMC13716215; doi:10.1371/journal.pone.0361401)
Supplement: Supplementary file 1 [file pone.0361401.s002.docx]

**Submission ID:** PONE-D-26-31145

**Original Article Title:** “IV-PAFNet: Infrared-visible cross-modal alignment and fusion network for nighttime UAV object detection”

**To:** PLOS ONE Editor

**Re:** Response to reviewers

Dear Editor,

Thank you for allowing us to submit a revised version of our manuscript and for providing us with the opportunity to address the comments from the Academic Editor and reviewers.

We are uploading (a) our point-by-point response to the comments below (Response to Reviewers), (b) an updated manuscript with changes marked (Revised Manuscript with Track Changes), and (c) an updated manuscript without change markings (Manuscript).

Best regards

Bin Zhou

**Journal Requirements:**

**Q1:** Please ensure that your manuscript meets PLOS ONE's style requirements, including those for file naming. The PLOS ONE style templates can be found at 
https://journals.plos.org/plosone/s/file?id=wjVg/PLOSOne_formatting_sample_main_body.pdf and 
<https://journals.plos.org/plosone/s/file?id=ba62/PLOSOne_formatting_sample_title_authors_affiliations.pdf>

**A1:** *We sincerely thank the Editor for this reminder. We have carefully revised the manuscript to ensure compliance with the PLOS ONE style requirements and formatting guidelines. The manuscript has been prepared and submitted in PDF format, and the file naming has also been checked and revised in accordance with the journal requirements. We have additionally reviewed the formatting of the title page, author information, tables, figures, equations, references, and other manuscript elements following the PLOS ONE formatting templates.*

*For example , Page 1- 3*

|  |  |  |
| --- | --- | --- |

**Q2:** Please note that PLOS One has specific guidelines on code sharing for submissions in which author-generated code underpins the findings in the manuscript. In these cases, we expect all author-generated code to be made available without restrictions upon publication of the work. Please review our guidelines at https://journals.plos.org/plosone/s/materials-and-software-sharing#loc-sharing-code and ensure that your code is shared in a way that follows best practice and facilitates reproducibility and reuse.

**A2:** *We fully agree with this requirement. At the current revision stage, we have made the testing and evaluation code publicly available through our project repository. Specifically, please refer to the code link at the end of the summary.*

*Page 1, Abstract*

**Q3:** Thank you for stating the following in the Acknowledgments Section of your manuscript: 
“The research is supported by the Natural Science Foundation of Shandong Province under Grant No. ZR2021MF031.”
We note that you have provided funding information that is not currently declared in your Funding Statement. However, funding information should not appear in the Acknowledgments section or other areas of your manuscript. We will only publish funding information present in the Funding Statement section of the online submission form. 
Please remove any funding-related text from the manuscript and let us know how you would like to update your Funding Statement. Currently, your Funding Statement reads as follows: 
“The research is supported by the Natural Science Foundation of Shandong Province under Grant No. ZR2021MF031.”
Please include your amended statements within your cover letter; we will change the online submission form on your behalf.

**A3:** *We have removed funding-related text from the manuscript body. We clearly stated the funding statement in the cover letter as follows: “This work was supported by the Natural Science Foundation of Shandong Province under Grant No. ZR2021MF031.”*

**Q4:** Thank you for stating the following financial disclosure: 
“The research is supported by the Natural Science Foundation of Shandong Province under Grant No. ZR2021MF031.”
Please state what role the funders took in the study.  If the funders had no role, please state: "The funders had no role in study design, data collection and analysis, decision to publish, or preparation of the manuscript." 
If this statement is not correct you must amend it as needed. 
Please include this amended Role of Funder statement in your cover letter; we will change the online submission form on your behalf.

**A4:** *We confirm that the funders provided financial support only and had no role in the scientific conduct or publication decisions of the study. We clearly stated the funding statement in the cover letter as follows: “The funders had no role in study design, data collection and analysis, decision to publish, or preparation of the manuscript.”*

**Q5:** We note that Figures 1 and 2 in your submission contain copyrighted images. All PLOS content is published under the Creative Commons Attribution License (CC BY 4.0), which means that the manuscript, images, and Supporting Information files will be freely available online, and any third party is permitted to access, download, copy, distribute, and use these materials in any way, even commercially, with proper attribution. For more information, see our copyright guidelines: <http://journals.plos.org/plosone/s/licenses-and-copyright>.
We require you to either (1) present written permission from the copyright holder to publish these figures specifically under the CC BY 4.0 license, or (2) remove the figures from your submission:

1) You may seek permission from the original copyright holder of Figure(s) [#] to publish the content specifically under the CC BY 4.0 license. 
We recommend that you contact the original copyright holder with the Content Permission Form (http://journals.plos.org/plosone/s/file?id=7c09/content-permission-form.pdf) and the following text:
“I request permission for the open-access journal PLOS ONE to publish XXX under the Creative Commons Attribution License (CCAL) CC BY 4.0 (http://creativecommons.org/licenses/by/4.0/). Please be aware that this license allows unrestricted use and distribution, even commercially, by third parties. Please reply and provide explicit written permission to publish XXX under a CC BY license and complete the attached form.”
Please upload the completed Content Permission Form or other proof of granted permissions as an "Other" file with your submission. 
In the figure caption of the copyrighted figure, please include the following text: “Reprinted from [ref] under a CC BY license, with permission from [name of publisher], original copyright [original copyright year].”
2) If you are unable to obtain permission from the original copyright holder to publish these figures under the CC BY 4.0 license or if the copyright holder’s requirements are incompatible with the CC BY 4.0 license, please either i) remove the figure or ii) supply a replacement figure that complies with the CC BY 4.0 license. Please check copyright information on all replacement figures and update the figure caption with source information. If applicable, please specify in the figure caption text when a figure is similar but not identical to the original image and is therefore for illustrative purposes only.

**A5:** *We sincerely thank the Editor for identifying this copyright issue. We have carefully revised these figures to comply with the licensing requirements of PLOS ONE. For Figure 1, the original images have been replaced with representative samples from the 3D-Net dataset, which is distributed under the Creative Commons Attribution 4.0 International (CC BY 4.0) license. The file name of a pair of sample images used in Figure 1 is "hausern_20_01_26_aufnahme5_017946.jpg", and the figure caption has been revised to clearly state the source of the images, the applicable CC BY 4.0 license, and that the samples are used only for illustration purposes. For Figure 2, because we were unable to obtain the copyright permission required for publication under the CC BY 4.0 license, we removed the figure from the revised manuscript. These changes ensure that the remaining figure content complies with the copyright and licensing requirements of PLOS ONE.*

*Page 7,Fig 1*

**Q6:** If the reviewer comments include a recommendation to cite specific previously published works, please review and evaluate these publications to determine whether they are relevant and should be cited. There is no requirement to cite these works unless the editor has indicated otherwise.

**A6:** *We have carefully reviewed the reviewer comments and did not find any requirement to cite specific additional publications. Therefore, we retained references that are directly relevant to the scientific background, methodology, experimental comparison, and evaluation protocol, and did not add unrelated citations merely for the purpose of revision.*

**Additional Editor Comments:**

**Q1:** More experimental results and analysis are required to fully support the main conclusions of this work.

**A1:** We sincerely thank the Academic Editor for this important comment. We agree that the original manuscript did not provide sufficiently detailed experimental evidence to fully support all mechanism-level claims. In the revised manuscript, we substantially strengthened the experimental section in four ways. First, we added fine-grained component-level ablation studies for ISA and MSFA, so that the effects of infrared purification, visible-guided alignment, channel recalibration, and spatial reliability modeling are analyzed separately. Second, we added statistical analysis using three random seeds and reported mean and standard deviation values to quantify robustness against random initialization. Third, we added computational complexity and inference-efficiency measurements, including parameter counts, FLOPs, peak memory, latency, and FPS. Fourth, we expanded the Discussion section to provide a balanced interpretation of benefits, limitations, and future directions. These additions directly address the concern that more experimental results and analysis are needed.

*Page 12, Fine-Grained Component Ablation*

*Page 12, Statistical Analysis*

*Page 12,Table 3*

*Page 12, Table 4*

*Page 12, Table 5*

*Page 14, Computational Complexity Analysis*

*,*

*Page 15, Table 6*

*Page 15, Discussion*

*Page 16, Conclusion*

**Q2:** Supplementary explanations for the replacement of the Camera-vehicle dataset from the corresponding author.

**A2:** Camera-vehicle dataset is used for generalization experiments. Due to the issue with the source of Camera-vehicle dataset and the fact that the research background of this paper is based on night and low-visibility scenarios, in this revision, we have customized Camera-vehicle1 dataset to replace Camera-vehicle dataset. The revised manuscript provides a detailed description of the Camera-vehicle1 dataset, and we have uploaded it to the github website for download and testing.

*Page 10, Datasets and Annotation Protocol*

*Page 16, Data Availability Statement*

**Review Comments to the Author**

**Reviewer #1**

**Q1:** Has the statistical analysis been performed appropriately and rigorously?

**A1:** We sincerely appreciate the reviewer for emphasizing the importance of statistical rigor. We agree that the original manuscript mainly reported point estimates and did not sufficiently quantify variability caused by random initialization. To address this concern, we added a Statistical Analysis subsection in the revised manuscript. We therefore independently trained the MMPedestron baseline and IV-PAFNet using seeds 42, 43, and 44 and report mAP@0.5:0.95 as the mean ± sample standard deviation over the three runs. MMPedestron obtains 61.98%, 62.44%, and 61.60% mAP@0.5:0.95, corresponding to 62.01 ± 0.42%, whereas IV-PAFNet obtains 62.63%, 63.59%, and 62.22% mAP@0.5:0.95, corresponding to 62.81 ± 0.70%. IV-PAFNet exceeds the baseline for every evaluated seed and improves mean mAP@0.5:0.95 by 0.80 percentage points.

*Page 12, Statistical Analysis*

*Page 12,Table 3*

**Q2:** Have the authors made all data underlying the findings in their manuscript fully available?

**A2:** We sincerely thank the reviewer for this important comment regarding data availability. We agree that all data and supporting materials underlying the findings of this study should be fully accessible. In the revised manuscript, we have explicitly reported the dataset split, horizontal-box conversion protocol, training and evaluation settings, and the individual mAP@0.5:0.95 values from the three random-seed runs, so that the key data underlying the reported results are directly available in the manuscript. In addition, all datasets used in this study have been made publicly available through a dedicated GitHub dataset repository. Supporting experimental materials, including the testing and evaluation code, configuration files, random-seed settings, and evaluation instructions, are provided through a separate project repository. At the current revision stage, the testing and evaluation code is publicly available; upon acceptance of the manuscript, we will release the complete code, including the training code, preprocessing scripts, model configurations, trained checkpoints, and detailed reproduction instructions.

*Page 1, Abstract*

*Page 10, Implementation Details and Evaluation Metrics*

*Page 16, Data Availability Statement*

**Q3:** Lines 176–190: The manuscript claims that ISA corrects RGB-infrared spatial misalignment, but it does not directly measure alignment quality. The reported mAP improvement shows better detection performance, but it does not prove that the proposed offset prediction and infrared resampling actually improve cross-modal alignment.

**A3:** We sincerely thank the reviewer for pointing out this important distinction. We fully agree that downstream detection mAP@0.5:0.95 is not a direct geometric registration metric and should not be used as sole proof that exact cross-modal alignment has been achieved. In response, we carefully revised the wording throughout the manuscript. We no longer claim that ISA “corrects” or “proves” exact RGB-infrared alignment. Instead, we describe the visible-guided resampling operation as a mechanism that alleviates local cross-modal spatial discrepancy and reduces the effect of modality inconsistency. To further clarify the role of the alignment component, we added a component-level ISA ablation. The MMPedestron baseline obtains 61.98% mAP@0.5:0.95, purification only obtains 58.82% mAP@0.5:0.95, alignment only obtains 60.63% mAP@0.5:0.95, and full ISA obtains 62.19% mAP@0.5:0.95. These results show that infrared purification and visible-guided resampling are more effective when used sequentially than when each component is applied independently. We explicitly interpret these results as downstream task-level evidence of complementarity, not as a direct geometric registration measurement.

*Page 6-8, Method, Infrared Signal Purification and Spatial Alignment*

*Page 12, Fine-Grained Component Ablation*

*Page 13, Table 4*

*Page 15, Discussion*

**Q4:** Lines 254–259: The ablation study is too coarse to support the mechanism-level claims. ISA combines infrared purification and spatial alignment, while MSFA combines channel-level and spatial-level fusion, but Table 3 only evaluates the complete ISA and MSFA modules, so the contribution of each subcomponent remains unclear.

**A4:** We sincerely thank the reviewer for this important comment. We agree that the original ablation study evaluated ISA and MSFA only at the module level and therefore did not sufficiently isolate the effects of their internal components. To address this issue, we added fine-grained component-level ablation experiments for both ISA and MSFA in the revised manuscript. For ISA, we separately evaluated infrared purification only, visible-guided alignment only, and the complete ISA. The purification-only and alignment-only variants achieve 58.82% and 60.63% mAP@0.5:0.95, respectively, whereas the complete ISA reaches 62.19%. These results show that neither operation provides a consistent improvement when used in isolation, while their sequential combination performs better than either individual variant and slightly exceeds the corresponding baseline. This indicates that infrared purification and visible-guided alignment are more effective when applied together rather than as isolated operations. For MSFA, we separately evaluated channel recalibration only, spatial reliability modeling only, and the complete MSFA. The channel-only variant achieves 60.30% mAP@0.5:0.95, while the spatial-only and complete MSFA variants both achieve 62.00%. However, the complete MSFA improves mAP@0.5 from 83.90% for the spatial-only variant to 85.50%, which is also higher than the 82.70% obtained by the channel-only variant. These results show that the two individual operations do not consistently improve all evaluation metrics when used separately, whereas their complete combination achieves the best mAP@0.5 among the MSFA variants while maintaining competitive mAP@0.5:0.95. Therefore, the revised ablation study supports our interpretation that the complete MSFA design is more effective when channel recalibration and spatial reliability modeling are used jointly rather than evaluated as isolated branches.

*Page 12, Fine-Grained Component Ablation*

*Page 12, Table 4*

*Page 12, Table 5*

**Q5:** Lines 221–253: The dataset and baseline protocols are not described in enough detail. Since the claims are made specifically on DroneVehicle and Camera-vehicle1, the manuscript should clearly state train/validation/test splits, annotation format, whether DroneVehicle oriented boxes were converted, and whether all baselines were retrained under the same protocol.

**A5:** We appreciate this important comment and have expanded the dataset and implementation descriptions. For DroneVehicle, the manuscript now states that the dataset contains 28,439 paired UAV-view visible and infrared images and that we follow the official split of 17,990 training pairs, 1,469 validation pairs, and 8,980 testing pairs. The original oriented bounding-box annotations are converted into their minimum enclosing horizontal rectangles because all compared detectors output horizontal bounding boxes, and the same horizontal-box evaluation protocol is applied throughout. All baseline detectors and IV-PAFNet are trained for 300 epochs using the same input resolution, data protocol, optimizer family, batch-size setting, EMA, AMP, and evaluation metrics. Camera-vehicle1 is retained as the original cross-scene benchmark: models trained on DroneVehicle are evaluated directly on its 12,483 paired images without target-domain fine-tuning. The rerun cross-scene inference yields 51.4% mAP@0.5:0.95 for IV-PAFNet.

*Page 10, Datasets and Annotation Protocol*

*Page 10, Implementation Details and Evaluation Metrics*

**Q6:** Lines 270–279: The paper targets UAV object detection but reports no computational-cost analysis. Since ISA and MSFA add filtering, gating, offset prediction, bilinear sampling, and attention, the manuscript should report parameters, FLOPs, memory, and inference latency/FPS to judge whether the accuracy gain is practically meaningful.

**A6:** We completely agree with the reviewer that computational cost is essential for evaluating UAV-oriented detection systems. In the revised manuscript, we added a dedicated Computational Complexity Analysis section. The baseline contains 43 M parameters, requires 125 G FLOPs, uses 3.4 GiB peak inference memory, and runs at 26 FPS with a latency of 38.46 ms. IV-PAFNet contains 52 M parameters, requires 154 G FLOPs, uses 3.8 GiB peak inference memory, and runs at 23 FPS with a latency of 43.48 ms. The measurements were conducted on an NVIDIA RTX 5090 GPU using a paired 640 × 640 visible-infrared input and batch size 1. We also specify that peak memory denotes the maximum allocated CUDA memory during inference and that latency includes forward propagation and model-specific post-processing but excludes image loading, disk I/O, and visualization. These results indicate a moderate computational trade-off rather than a lightweight design. In addition, we added a Discussion section noting that ISA and MSFA introduce additional parameters, FLOPs, memory use, and latency; although IV-PAFNet shows a slight decrease in FPS compared with the MMPedestron baseline, it achieves a competitive advantage in detection accuracy.

*Page 14, Computational Complexity Analysis*

*Page 15, Table 6*

*Page 15, Discussion*

**Q7:** Lines 280–283: Reproducibility is incomplete. The paper provides dataset links but not source code, training configurations, split files, random seeds, checkpoints, or evaluation scripts, even though the main contribution is an architectural method.

**A7:** We sincerely thank the reviewer for emphasizing the importance of reproducibility. We agree that the original manuscript did not provide sufficient materials to independently reproduce the reported experiments. In the revised manuscript, we have explicitly reported the official dataset splits, horizontal-box conversion protocol, input resolution, number of training epochs, batch size, optimizer, learning-rate schedule, EMA and AMP settings, evaluation metrics, and random seeds (42, 43, and 44), and the individual mAP@0.5:0.95 values from the three random-seed runs are provided in Table 3. The datasets used in this study are publicly available through a dedicated GitHub dataset repository. At the current revision stage, the testing and evaluation code is publicly available through a separate project repository. Upon acceptance of the manuscript, we will further release the complete source code, training configuration files, preprocessing scripts, trained checkpoints, and detailed reproduction instructions. The complete code and associated materials will be made publicly accessible without restrictions no later than publication.

*Page 1, Abstract*

*Page 10, Datasets and Annotation Protocol*

*Page 10, Implementation Details and Evaluation Metrics*

*Page 16, Data Availability Statement*

**Reviewer #2**

**Q1:** This paper does not contain the Discussion section. I would suggest adding the Discussion section where authors interpret the results, explain their significance, compare them with previous research, acknowledge limitations, and describe implications.

**A1:** We sincerely thank the reviewer for this constructive suggestion. We agree that the original manuscript lacked a dedicated section for interpreting the experimental findings. In the revised manuscript, we added a new Discussion section. This section provides a more balanced interpretation of the component-level ablation results for ISA and MSFA, including the combined effects of infrared purification and visible-guided resampling in ISA and the different behaviors of channel recalibration and spatial reliability modeling in MSFA. It also discusses the statistical results obtained from three random seeds and the computational trade-off introduced by the proposed modules in terms of parameters, FLOPs, memory use, latency, and FPS. These additions provide a clearer and more cautious interpretation of the experimental results.

*Page 15, Discussion*
