# Supplementary material for: IV-PAFNet: Infrared-visible cross-modal alignment and fusion network for nighttime UAV object detection
Source: PLoS One. 2026 Oct 28;21(10):e0361401. doi: 10.1371/journal.pone.0361401 (PMC13716215; doi:10.1371/journal.pone.0361401)
Supplement: Supplementary file 2 [file pone.0361401.s003.docx]

**Submission ID:** PONE-D-26-31145R1

**Original Article Title:** “IV-PAFNet: Infrared-visible cross-modal alignment and fusion network for nighttime UAV object detection”

**To:** PLOS ONE Editor

**Re:** Response to reviewers

Dear Editor,

Thank you for allowing us to submit a revised version of our manuscript and for providing us with the opportunity to address the comments from the Academic Editor and reviewers.

We are uploading (a) our point-by-point response to the comments below (Response to Reviewers), (b) an updated manuscript with changes marked (Revised Manuscript with Track Changes), and (c) an updated manuscript without change markings (Manuscript).

Best regards

Bin Zhou

**Journal Requirements:**

**Q1:** If applicable, we recommend that you deposit your laboratory protocols in protocols.io to enhance the reproducibility of your results. Protocols.io assigns your protocol its own identifier (DOI) so that it can be cited independently in the future. For instructions see: <https://journals.plos.org/plosone/s/submission-guidelines#loc-laboratory-protocols>. Additionally, PLOS One offers an option for publishing peer-reviewed Lab Protocol articles, which describe protocols hosted on protocols.io. Read more information on sharing protocols at [https://plos.org/protocols?utm_medium=editorial-email&utm_source=authorletters&utm_camp aign=protocols](https://plos.org/protocols?utm_medium=editorial-email&utm_source=authorletters&utm_campaign=protocols).

As the corresponding author, your ORCID iD is verified in the submission system and will appear in the published article. PLOS supports the use of ORCID, and we encourage all coauthors to register for an ORCID iD and use it as well. Please encourage your coauthors to verify their ORCID iD within the submission system before final acceptance, as unverified ORCID iDs will not appear in the published article. Only the individual author can complete the verification step; PLOS staff cannot verify ORCID iDs on behalf of authors.

**A1:** *We sincerely thank the Editor for this reminder. The present study does not introduce a laboratory protocol requiring a separate protocols.io deposit. We have noted the ORCID guidance and the corresponding author will verify an ORCID iD (*[*https://orcid.org/0000-0002-0314-2595*](https://orcid.org/0000-0002-0314-2595)*) in the submission system before final acceptance.*

**Q2:** If the reviewer comments include a recommendation to cite specific previously published works, please review and evaluate these publications to determine whether they are relevant and should be cited. There is no requirement to cite these works unless the editor has indicated otherwise. Please review your reference list to ensure that it is complete and correct. If you have cited papers that have been retracted, please include the rationale for doing so in the manuscript text, or remove these references and replace them with relevant current references. Any changes to the reference list should be mentioned in the rebuttal letter that accompanies your revised manuscript. If you need to cite a retracted article, indicate the article’s retracted status in the References list and also include a citation and full reference for the retraction notice.

**A2:** *We sincerely thank the Editor for this reminder. We reviewed the reference list for completeness and bibliographic accuracy, added DOI information where available, and checked the cited publications for retraction status. No cited article was identified as retracted, and the reviewers did not request the addition of any specific publication in this review round.*

**Review Comments to the Author**

**Reviewer #1**

The manuscript is within the scope of PLOS ONE because it presents original research on infrared-visible UAV object detection and evaluates the proposed method on publicly available datasets. However, I recommend Major Revision because the reproducibility of the main findings remains insufficiently supported.

**Q1:** Lines 290-292 (Data Availability Statement): The primary conclusion of the manuscript is that IV-PAFNet achieves superior detection performance relative to the evaluated baselines. However, the complete implementation required to reproduce these findings, including training code, model checkpoints, preprocessing scripts, configuration files, and evaluation scripts, is not currently available. This limits independent verification of the reported results.

**A1:** We sincerely thank the reviewer for emphasizing the importance of reproducibility. We have now made the training and evaluation entry points, paired infrared-visible data loader, preprocessing and DroneVehicle annotation-conversion scripts, IV-PAFNet implementation, 73-epoch configurations for seeds 42, 43, and 44, component configurations, environment specifications, and step-by-step commands publicly available. The released training procedure writes checkpoints, and the evaluation command consumes a saved checkpoint directly. The source repository is available at <https://github.com/iv-pafnet/IV-PAFNet>, while dataset preparation information, checksums, and versioned files are available at <https://github.com/iv-pafnet/IV-PAFNet_dataset>. We also verified model construction with a weight-free forward test and verified the released training path with a small end-to-end smoke test covering data loading, forward and backward propagation, optimization, validation, and checkpoint writing.

***Page 17, Data Availability Statement***

**Q2:** Lines 186-195 and Tables 1-6: The manuscript provides additional implementation details, dataset protocols, and random-seed information. Nevertheless, the information available at the time of review does not appear sufficient to fully reproduce the reported experiments and performance values. For a study whose main contribution is a novel architectural design, reproducibility is particularly important.

**A2:** We sincerely thank the reviewer for this important comment. The public README now maps the full IV-PAFNet model, controlled dual-stream baseline, and component variants to their dedicated configuration files and commands. The manuscript and release consistently specify 73 training epochs, input size, optimizer, learning-rate schedule, exponential moving average, mixed-precision setting, global batch size, evaluated seeds, and the rule used to select the single-seed results. The release further documents dataset conversion, expected directory layouts, checkpoint creation, and checkpoint-based evaluation. These materials connect the reported experiments and tables to executable configurations and commands.

***Page 10, Implementation Details and Evaluation Metrics***

**Q3:** Lines 216-220: The reported improvement over the strongest baseline is relatively modest (62.81% versus 62.01% mAP@0.5:0.95 on DroneVehicle). In such cases, access to the complete experimental framework is important for confirming the robustness and repeatability of the reported performance differences.

**A3:** We sincerely thank the reviewer for highlighting this point. We agree that the 0.80-percentage-point mean difference should be considered together with repeated-run evidence and access to the experimental framework. The revised manuscript reports results for seeds 42, 43, and 44: IV-PAFNet achieves 62.81 ± 0.70% mAP@0.5:0.95, while the controlled dual-stream baseline achieves 62.01 ± 0.42%. The public repository provides the corresponding seed-specific configurations, training entry point, and checkpoint-based evaluation procedure. The Discussion describes this difference as measured but modest and limits the statistical interpretation to the reported three-seed comparison.

***Page 12, Statistical Analysis and Table 3***

**Q4:** Response to Reviewer Q7: The authors state that the complete code, trained checkpoints, and additional materials will be released upon acceptance. While this commitment is appreciated, the manuscript's principal conclusions are based on comparative performance claims. Providing the materials necessary to reproduce these results would substantially strengthen the evidence supporting the study.

**A4:** We sincerely thank the reviewer for this important comment. We agree that the reproducibility materials should be available during review rather than only after acceptance. The source code, configurations, environment files, and step-by-step training and evaluation instructions are now publicly available at <https://github.com/iv-pafnet/IV-PAFNet>. Dataset access, preparation details, checksums, and versioned release files are publicly available at <https://github.com/iv-pafnet/IV-PAFNet_dataset>. We therefore replaced the previous "available upon acceptance" statement with these direct repository links in the Data Availability Statement.

***Page 17, Data Availability Statement***

**Reviewer #2**

**Q1:** I would suggest expanding the description of the study limitations in the "Discussion" section. Thanks!

**A1:** We sincerely thank the reviewer for this constructive suggestion. We have expanded the Discussion to clarify the scope of the present evaluation and the most relevant extensions. The revised text clarifies that both DroneVehicle and Camera-vehicle1 contain paired infrared-visible images while covering complementary viewpoints and backgrounds, states the synchronized-pair assumption, and identifies sensor dropout, synchronization error, calibration change, and thermal crossover as useful stress-test conditions. It also distinguishes downstream detection evaluation from direct geometric alignment measurement, summarizes the three-seed evidence, and clarifies that the reported 23 FPS measures model inference rather than complete onboard-system throughput. The revised Discussion further identifies controlled spatial perturbations, missing or corrupted modalities, additional multispectral datasets and repetitions, and measurements on target UAV hardware as future evaluation directions.

***Pages 15-16, Discussion***
